# Noninvasive Electromagnetic Phrenic Nerve Stimulation in Critically Ill Patients: A Feasibility Study

**DOI:** 10.1016/j.chest.2024.02.035

**Published:** 2024-02-24

**Authors:** Alessandro Panelli, Aline M. Grimm, Sven Krause, Michael A. Verfuß, Bernhard Ulm, Julius J. Grunow, Hermann G. Bartels, Niklas M. Carbon, Thomas Niederhauser, Steffen Weber-Carstens, Laurent Brochard, Stefan J. Schaller

**Affiliations:** a Charité – Universitätsmedizin Berlin, corporate member of Freie Universität Berlin and Humboldt-Universität zu Berlin, Department of Anesthesiology and Intensive Care Medicine (CCM/CVK), Berlin, Germany; b Technical University of Munich, School of Medicine, Klinikum rechts der Isar, Department of Anesthesiology and Intensive Care, Munich, Germany; c Department of Anaesthesiology and Intensive Care Medicine, School of Medicine, University of Ulm, Ulm, Germany; d Department of Anesthesiology, Friedrich-Alexander-Universität Erlangen-Nürnberg, Uniklinikum Erlangen, Erlangen, Germany; e Institute for Human Centered Engineering, Bern University of Applied Sciences, Biel/Bienne, Switzerland; f Keenan Research Centre for Biomedical Science, Li Ka Shing Knowledge Institute, Unity Health Toronto, Toronto, ON, Canada; g Interdepartmental Division of Critical Care, University of Toronto, Toronto, ON, Canada

**Keywords:** critical care, critical illness, ICUs, interactive ventilatory support, magnetic field therapy, magnetic fields, magnetic stimulation therapy, muscular atrophy, respiration, artificial, ventilator weaning

## Abstract

**Background:**

Electromagnetic stimulation of the phrenic nerve induces diaphragm contractions, but no coils for clinical use have been available. We recently demonstrated the feasibility of ventilation using bilateral transcutaneous noninvasive electromagnetic phrenic nerve stimulation (NEPNS) before surgery in lung-healthy patients with healthy weight in a dose-dependent manner.

**Research Question:**

Is NEPNS feasible in critically ill patients in an ICU setting?

**Study Design and Methods:**

This feasibility nonrandomized controlled study aimed to enroll patients within 36 h of intubation who were expected to remain ventilated for ≥ 72 h. The intervention group received 15-min bilateral transcutaneous NEPNS bid, whereas the control group received standard care. If sufficient, NEPNS was used without pressure support to ventilate the patient; pressure support was added if necessary to ventilate the patient adequately. The primary outcome was feasibility, measured as time to find the optimal stimulation position. Further end points were sessions performed according to the protocol or allowing a next-day catch-up session and tidal volume achieved with stimulation reaching only 3 to 6 mL/kg ideal body weight (IBW). A secondary end point was expiratory diaphragm thickness measured with ultrasound from days 1 to 10 (or extubation).

**Results:**

The revised European Union regulation mandated reapproval of medical devices, prematurely halting the study. Eleven patients (five in the intervention group, six in the control group) were enrolled. The median time to find an adequate stimulation position was 23 s (interquartile range, 12-62 s). The intervention bid was executed in 87% of patients, and 92% of patients including a next-day catch-up session. Ventilation with 3 to 6 mL/kg IBW was achieved in 732 of 1,701 stimulations (43.0%) with stimulation only and in 2,511 of 4,036 stimulations (62.2%) with additional pressure support. A decrease in diaphragm thickness was prevented by bilateral NEPNS (*P* = .034) until day 10.

**Interpretation:**

Bilateral transcutaneous NEPNS was feasible in the ICU setting with the potential benefit of preventing diaphragm atrophy during mechanical ventilation. NEPNS ventilation effectiveness needs further assessment.

**Trial Registry:**

ClinicalTrials.gov; No.: NCT05238753; URL: www.clinicaltrials.gov

**Take-home Points**

**Study Question:** Is bilateral transcutaneous noninvasive electromagnetic phrenic nerve stimulation (NEPNS) feasible in the critical care setting?

**Results:** The deployment demonstrated a favorable speed, with a mean time of 23 s and a high realization rate of 92%. Furthermore, 81% of tidal volumes delivered through pressure support ventilation-assisted NEPNS fell within the ultra-lung-protective spectrum of 3 to 6 mL/kg ideal body weight.

**Interpretation:** This study demonstrates that bilateral transcutaneous NEPNS is feasible in the ICU.

Diaphragm atrophy, which increases the risk of weaning failure by approximately 20%,^1^^,^^2^ rapidly develops within 18 to 69 h of invasive mechanical ventilation.^3^, ^4^, ^5^ To prevent atrophy via stimulated contractions, as evidenced by previous molecular biology studies,^6^^,^^7^ diaphragm activation can be attained through either phrenic nerve or direct diaphragm stimulation; both invasive and noninvasive methods can be used for these approaches.^8^ In this context, bilateral transcutaneous noninvasive electromagnetic phrenic nerve stimulation (NEPNS) using tailored phrenic nerve coils has been proposed as a potential alternative to invasive phrenic nerve stimulation methods. Bilateral transcutaneous NEPNS can mimic physiologic breathing cycles, leading to diaphragm contraction, thereby potentially mitigating the onset of diaphragm atrophy. In one previous study, NEPNS in nonanesthetized patients was initiated using transcranial magnetic stimulation coils, presenting limitations including the usability of cumbersome coils, device synchronization issues, and volitional components from stimulating awake volunteers.^9^ We performed bilateral transcutaneous NEPNS for the first time, using newly designed compact coils specifically designed for NEPNS, in lung-healthy anesthetized patients in the operating room, where any volitional element could be avoided. We demonstrated that this technique could be used to mimic spontaneous breathing in anesthetized intubated patients, generating tidal volumes (TVs) in the lung protective range.^10^ The feasibility and efficacy of bilateral NEPNS in critically ill patients remain unexplored.

Therefore, the present study aimed to test the feasibility of bilateral transcutaneous NEPNS in critically ill patients by repetitively stimulating the phrenic nerves, generating diaphragm contraction noninvasively, and determining the success rate of bilateral transcutaneous NEPNS administration. This experimental protocol aimed primarily to assess the speed of deployment and adherence to planned intervention within the ICU setting and to investigate the possibility of achieving adequate TV in critically ill patients. We hypothesized that diaphragm contraction achieved through bilateral transcutaneous NEPNS is feasible in the ICU setting and could be a prophylaxis for diaphragm atrophy.

## Study Design and Methods

The ethics committee of Charité—Universitätsmedizin Berlin granted ethical approval (Identifier: EA2/258/21) on December 8, 2021, for this STIMIT II study. The study was a prospective open-label, nonrandomized study performed in one medical center. Informed consent was obtained from the patient or legal representative.

### Eligibility Criteria

This feasibility study aimed to include 30 adult patients admitted to an ICU within 36 h of intubation who were expected to be ventilated for at least 72 h. Exclusion criteria are presented in e-Appendix 1.

### Conduct of the Study

The initial study design (ClinicalTrials.gov Identifier: NCT05238753) aimed at different stimulation training protocols: bid, tid, or five times per day until extubation or day 10. However, after the mandatory reapproval of medical devices in the European Union (renewed European Union law 2017/745), only a bid timing could be executed for this training protocol, and the data presented herein are based solely on this group.

To gather the necessary data, the intervention group (n = 5) received 15 min of bilateral transcutaneous NEPNS sessions bid, whereas the control group (n = 6) was not stimulated. Fifteen minutes of NEPNS were delivered as three 5-min NEPNS sessions of training, separated by at least 1 min of pause, during which the patient was ventilated again using the same mechanical ventilation mode previously set by the treatment team.

The setup is presented in e-Figure 1. To identify coil positioning for stimulation (capture point [CP]), the phrenic nerve was localized according to anatomic landmarks. Contractions were validated through diaphragm ultrasound and ventilator flow adjustments. Further details on CP establishment and stimulation maintenance are provided in e-Appendix 1. The bilateral transcutaneous NEPNS stimulator specifications were equivalent to those published before (e-Appendix 1).^10^

TV, capture loss during the session, and presence or absence of sternocleidomastoid muscle or plexus brachialis costimulations were documented. Additionally, before every stimulation session, vital signs, clinical frailty score, Charlson Comorbidity Index, Acute Physiology and Chronic Health Evaluation II score, Sequential Organ Failure Assessment score, worst Glasgow Coma Scale score, Richmond Agitation Sedation Scale score, and COVID-19 polymerase chain reaction results were documented. Transpulmonary pressure (P_L_), airway pressure (maximal and minimal tracheal pressure), occlusion pressure (P_occ_),^11^ flow, and volume were recorded and analyzed; the hardware used is described in e-Appendix 1. Patients in the control group received standard of care without any stimulations. Diaphragm ultrasound measurements were obtained in both groups from days 1 through 10 (or until extubation). The description of the ultrasound assessment and its masked analysis are presented in e-Appendix 1.

### Primary and Secondary End Points

The primary outcome was the feasibility and efficacy of bilateral transcutaneous NEPNS in critically ill patients. The primary end point was NEPNS deployment speed (time to find CP). Key secondary end points were therapeutic protocol adherence and expiratory diaphragm thickness (Tdi_exp_) from days 1 through 10 (or extubation); if a scheduled session was not performed according to the protocol, a next-day catch-up session the following day was allowed. Additional secondary end points were the descriptive analysis of breathing mechanics during NEPNS (TV, transpulmonary pressure, tracheal airway pressures, driving pressure, lung compliance, lung resistance, and occlusion pressure).

Regarding safety, the STIMIT II study was an investigator-initiated study according to § 47 Medical Devices Law of German legislation (Medizinprodukte-Durchführungsgesetz). Device incidents had to be documented and reported according to German medical devices operator regulation (Medizinprodukte-Betreiberverordnung). Serious adverse events were recorded only if they were possibly related to the intervention during the intervention itself.

### Statistical Analysis

Because this was a feasibility study and, to our knowledge, the first application of bilateral transcutaneous NEPNS in critically ill patients, no sample size was calculated and 30 patients were planned. Descriptive statistical analysis was performed using mean ± SD or median (interquartile range [IQR]), as appropriate. Absolute and relative frequencies were used for categorical variables.

For the primary end point feasibility, only a descriptive statement was used. For the secondary end points, a Mann-Whitney *U* test was used for continuous variables, depending on distribution, and the Fisher exact test for categorical variables. The Wilcoxon signed-rank test was used to compare dependent samples in nonparametric data. For expiratory diaphragm thickness, a mixed model with the factors group, time, and their interaction term was used.

A *P* value of .05 was used as significant. Statistical analysis was performed using IBM SPSS Statistics version 27.0 (SPSS, Inc.), and figures were created with the same software or R version 4.1.1 software (R Foundation for Statistical Computing).

## Results

The study was conducted between January 21 and May 15, 2022, and was stopped prematurely because the conformité européenne (CE) certificate of the coils was withdrawn because of the revised European Union regulation on medical devices. In compliance with legislation, the coils originally obtained CE certification through the magnetic stimulator company as an additional accessory of a CE-cleared system. However, because of changing regulations and additional costs associated, the CE certificate was revoked. The CE revocation did not occur because of safety concerns or other issues.

A total of 11 patients (five patients from the intervention group A and six patients from the control group) (e-Fig 2) were enrolled. The duration from intubation to initial stimulation was 21.3 ± 12.2 h. Demographics and ICU admission diagnoses are presented in Table 1.

**Table 1:** Demographics of Included Patients

Variable	Intervention Group (n = 5)	Control Group (n = 6)	All Patients (N = 11)
Age, y	55 (41-71)	73 (62-83)	69 (48-75)
Female sex	1 (20)	1 (17)	2 (18)
Height	175 (165-181)	173 (167-178)	175 (167-177)
Weight	77.0 (71.5-85.0)	74.5 (70.3-80.5)	75.0 (73.0-80.0)
BMI , kg/m^2^	26.1 (21.8-30.6)	25.6 (23.5-26.6)	26.1 (22.2-27.6)
CCI	3 (1-4)	3 (1-4)	3 (1-4)
APACHE II score at admission	20 (16-26)	20 (10-25)	20 (15-26)
SOFA score at admission	5 (3-10)	9 (4-10)	6 (4-9)
ICU admission diagnoses			
ARDS	3 (60)	3 (50)	6 (55)
Stroke	2 (40)	2 (33)	4 (36)
Sepsis	0 (0)	1 (17)	1 (9)

Data are presented as No. (%) or median (interquartile range). No group differences are present in the variables presented. APACHE = Acute Physiology and Chronic Health Evaluation; CCI = Charlson Comorbidity Index; SOFA = Sequential Organ Failure Assessment.

### Primary End Point

**Figure 1:**
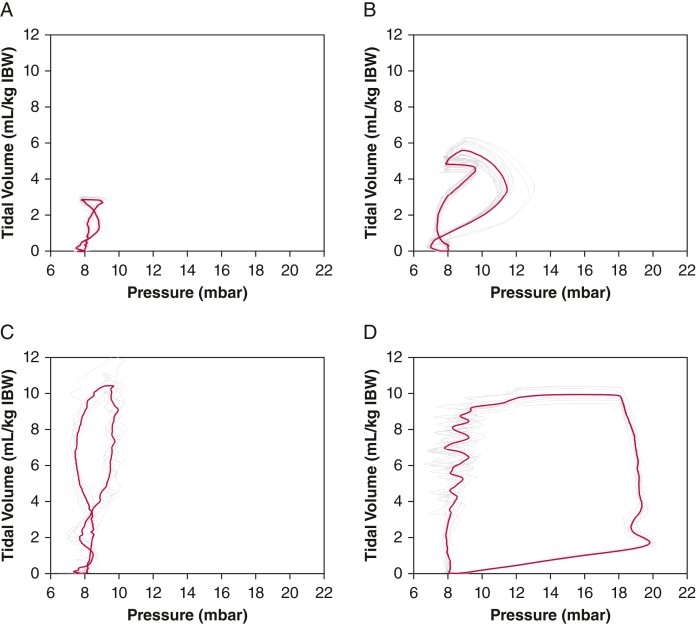
A-D, Example pressure-volume curves for bilateral noninvasive electromagnetic phrenic nerve stimulation (NEPNS) stimulated only, without pressure support ventilation (PSV) (A), bilateral NEPNS stimulation with PSV (B), bilateral NEPNS with spontaneous breathing (C), and during mechanical ventilation for comparison (D). Single breaths are depicted in grey; red curves represent the mean values over one stimulation session. IBW = ideal body weight; mbar = millibar.

**Figure 2:**
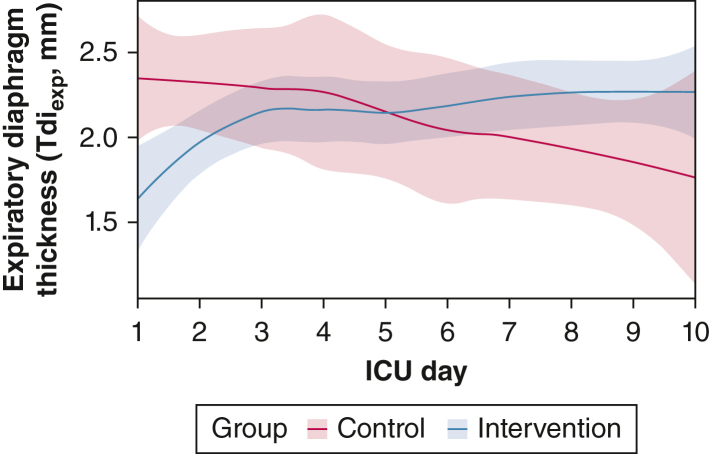
Graph showing trajectory of Tdi_exp_ (in millimeters) measured by ultrasound over time (with 95% CI) for intervention group (blue) and control group (red). The intervention group received transcutaneous bilateral noninvasive electromagnetic phrenic nerve stimulation bid for 15 min, which increased Tdi_exp_ compared with the control group as demonstrated in a mixed model analysis using Tdi_exp_ from days 1 through 10 (or until extubation). The intervention (*P* = .034), time (*P* = .032), and the interaction of time and group (*P* = .004) were significant. The intervention group underwent two measurements per day, and the control group underwent one measurement per day. Tdi_exp_ = expiratory diaphragm thickness.

The average time to find CP was 23 s (IQR, 12-62s) (Table 2). To reach the defined goal of ventilation with at least 3 mL/kg ideal body weight (IBW), stimulated breaths needed to be applied as: (1) stimulated-only breaths (goal reached in 45.7%), during which TVs were generated by bilateral NEPNS only, that is, without spontaneous breathing from the patient (Fig 1A); (2) stimulation with pressure support ventilation (PSV) support (goal reached in 81.0%), (Fig 1B); and (3) stimulation manually synchronized with spontaneous breathing (goal reached in 99.4%), during which the patient performed breaths spontaneously, which were supported with manually synchronized bilateral NEPNS (Fig 1C). TVs achieved are presented in Table 3 and e-Table 1.

**Table 2:** Outcomes in the Stimulated Group

Outcome	Data
Frequency of nonfeasible stimulation sessions:	
Because of organizational or patient-related reasons	13/100 (13)
With a next-day catch-up session	8/100 (8)
Time to find capture, s	23 (12-62)

Data are presented as No./Total No. (%) or median (interquartile range).

**Table 3:** Tidal Volumes Achieved During Bilateral NEPNS Without PSV, With PSV, and During Spontaneous Breathing

Stimulation Type	Tidal Volume, mL/kg IBW	Bilateral NEPNS Breaths			Tidal Volume, mL/kg IBW		
		No.	No. on a Single Mode	On Stimulations in a Single Mode, %	Median (IQR)	Minimum	Maximum
Bilateral NEPNS only (without PSV)	< 3	923	1,701	54.3	1.93 (1.09-2.48)	0.00	3.00
	3-6	732		43.0	3.87 (3.44-4.32)	3.00	6.00
	> 6	46		2.7	6.72 (6.38-7.21)	6.05	7.80
Bilateral NEPNS with PSV	< 3	768	4,036	19.0	2.74 (2.56-2.87)	0.00	3.00
	3-6	2,511		62.2	4.34 (3.46-5.44)	3.00	6.00
	> 6	757		18.8	7.26 (6.53-10.97)	6.00	20.14
Spontaneous breathing plus bilateral NEPNS	< 3	4	619	0.6	0.36 (0.28-0.39)	0.07	0.43
	3-6	0		0.0	0.00	0.00	0.00
	> 6	615		99.4	9.62 (8.62-10.73)	6.49	17.19
Total stimulations	…	6,356	6,356	…	…	…	…

Data are presented as No. unless otherwise indicated. IBW = ideal body weight; IQR = interquartile range; NEPNS = transcutaneous noninvasive phrenic nerve stimulation; PSV: pressure support ventilation.

### Secondary End Points

With respect to adherence to protocol, the intervention bid was executed in 87% of patients without and 92% of patients with a next-day catch-up session, respectively (Table 2). Patients in the intervention group received bilateral NEPNS for 27.9 ± 7.4 min/d over the course of 9.4 ± 1.3 stimulation days. The accumulated stimulation time per patient was 279.0 ± 47.0 min during the 10-day observation period.

Dynamic transpulmonary pressure swings (P_L_) during bilateral NEPNS were 11.2 millibar (mbar) (IQR, 9.3-13.0 mbar), 19.0 mbar (IQR, 12.9-24.5 mbar), and 23.0 mbar (IQR, 12.8-27.2 mbar) for bilateral NEPNS only, bilateral NEPNS with PSV, and bilateral NEPNS with spontaneous breathing, respectively. Bilateral NEPNS-stimulated maximal and minimal airway pressure for the intervention group were 8.8 mbar (IQR, 8.4-9.3 mbar) and 7.3 mbar (IQR, 6.1-7.5 mbar), respectively. Bilateral NEPNS-stimulated P_occ_ was 4.4 mbar (IQR, 3.0-9.9 mbar). Data on these parameters were not collected for the control group because bilateral NEPNS was not performed in those patients.

Lung compliance and lung resistance are presented in e-Table 2. No statistical difference between the groups was found for either. Tdi_insp_, Tdi_exp_, diaphragm thickening fraction, and diaphragm excursion are presented in e-Table 3. A signal of protection against diaphragmatic atrophy by NEPNS exists; this was confirmed in a mixed-model analysis using all Tdi_exp_ ultrasound measurements from days 1 through 10. Group (*P* = .034), time (*P* = .032), and the interaction of time and group (*P* = .004) were significant (Fig 2).

Exploratory clinical end points are available in e-Table 4. No device incidents were reported; adverse events are presented in e-Table 5. Ventilator modes, peak inspiratory pressure, positive end-expiratory pressure, Fio_2_, and ventilator-free days are presented in e-Table 6.

## Discussion

This study represents to our knowledge the first use of a noninvasive technique to stimulate phrenic nerves, generating diaphragm contraction in the critical care environment. A standardized bilateral transcutaneous NEPNS implementation in a clinical setting was feasible, demonstrated by a fast deployment time (< 1 min), an adherence of 92% to an a priori-defined treatment protocol, and reaching predefined goals of TVs.

Despite NEPNS being a decades-proven technique, cumbersome coils hindered continuous bilateral NEPNS.^9^ Only a few studies tested the new compact coils in lung-healthy patients undergoing general anesthesia, demonstrating that sufficient TVs through NEPNS in the perioperative setting were feasible.^10^^,^^12^ Mueller et al^12^ used the same stimulation hardware in lung-healthy patients undergoing elective orthopedic surgery. They achieved median TVs of up to 279 mL (range, 80-557 mL) with 40% stimulation intensity. Although TVs normalized to body weight were not provided, using the median weight of 75 kg, a value around 3.7 mL/kg body weight could be assumed. In our previous perioperative study, the median TV was 7.4 ± 3.1 mL/kg IBW.^10^ Both studies applied only brief stimulation for short periods, that is, prolonged diaphragm training was not investigated. Furthermore, both studies demonstrated safe volume and pressure levels within lung protection ranges of < 0 positive end-expiratory pressure conditions, which is unsuitable for critically ill patients.^10^^,^^12^

In this ICU study, three distinct patterns of bilateral transcutaneous NEPNS stimulation patterns were identified: (1) stimulation-only breaths (bilateral NEPNS only), (2) PSV-supported bilateral NEPNS, and (3) spontaneous breathing with manually synchronized bilateral NEPNS. Among these patterns, TVs between 3 and 6 mL/kg IBW range were achieved most frequently with PSV-supported bilateral NEPNS (39.5% of stimulated breaths). Conversely, during manually synchronized bilateral transcutaneous NEPNS in conjunction with spontaneous breathing, the frequency of 3 to 6 mL/kg IBW-achieved TVs was nearly 0% because patients autonomously regulated TVs after stimulation of > 6 mL/kg IBW in 99.4% of all stimulations. This raises the question of whether stimulation of patients during maintained spontaneous breathing per se makes sense or is necessary. Using bilateral transcutaneous NEPNS without PSV demonstrated inadequate TVs in patients during this stimulation type, with 54% of breaths decreasing to < 3 mL/kg IBW. Occasional TVs of > 8 mL/kg IBW were documented. Based on these findings, bilateral transcutaneous NEPNS by itself may not always maintain adequate minute ventilation in critically ill patients, but supporting it with PSV seems to be a treatment option for critically ill patients.

Comparisons with studies using diverse magnetic stimulators, coils, or other stimulation techniques are challenging because of inherent differences in the generated magnetic field shapes and intensities. These variations result in different effects on the phrenic nerve and diaphragm contractions, making direct comparisons limited, as previously discussed.^10^ All other techniques for phrenic nerve stimulation were classified systematically in a recent review into invasive electrical, noninvasive electrical, or noninvasive (electro)magnetic, summarizing the results and underlying benefits and disadvantages.^8^ Direct phrenic nerve stimulation during mechanical ventilation (MV) showed positive outcomes at the molecular level, including improved mitochondrial function and reduced oxidative stress.^6^^,^^7^ O’Rourke et al^13^ used percutaneous needle stimulation for spontaneous breathing support, akin to the third pattern we identified. They reported a significant 34.6 ± 16.9% increase in TVs for stimulated spontaneous breaths and increased diaphragm thickness by 15%. Diaphragm pacing in patients with spinal cord injury achieved successful weaning without tracheostomy in 69% of patients.^14^ Transvenous temporary diaphragm neurostimulation demonstrated significant improvements in maximum inspiratory pressure, indicating the potential for inhibiting diaphragmatic weakness during MV.^15^ The current expert consensus on lung-protective MV revolves around mitigating barotrauma through lower MV and transpulmonary pressures.^16^, ^17^, ^18^ Throughout the present experiments with bilateral transcutaneous NEPNS, transpulmonary pressures (maximum transpulmonary pressure [P_Lmax_] and minimum transpulmonary pressure [P_Lmin_]) consistently remained < 20 mbar.^19^, ^20^, ^21^ This adherence to the upper limit of the physiologic range (set at 15-20 mbar) further supports the feasibility of our intervention during stimulation only and PSV-supported stimulation.^21^ While during stimulation only, P_Lmax_ always stayed within the safety threshold, PSV-stimulated P_Lmax_ regularly exceeded this threshold and stimulation with spontaneous breathing in most patients. Therefore, caution in patients with spontaneous breathing is warranted.

Airway driving pressure during bilateral transcutaneous NEPNS remained at < 15 mbar excursions over positive end-expiratory pressure, which is considered the lung protective range of pressures,^22^ demonstrating a low risk of lung stress.^21^ Although airway driving pressure was safely within range during NEPNS, only transpulmonary pressure (P_L_) considers the positive pressure from the ventilator support and the negative pressure from NEPNS, considering that the primary parameter P_L_ is crucial to prevent lung overdistention. Stimulated (bilateral transcutaneous NEPNS) P_occ_ remained < 10 mbar threshold, suggesting a safe target for lung and diaphragm-protective MV.^23^

To summarize, observed low airway pressures (maximal and minimal airway pressure) and consequently low driving pressure during bilateral transcutaneous NEPNS, along with safe ranges for P_occ_, validate the findings of our earlier proof-of-concept study.^10^ These results reinforce NEPNS’s lung and diaphragm protective nature, operating within a low lung-stress pressure spectrum. A certain level of caution is advisable to prevent the risk of barotrauma during stimulation within the context of spontaneous breathing to ensure that P_Lmax_ is set at 15 to 20 mbar, as advocated by Goligher et al.^21^ Importantly, our study used manual stimulation. In the future, automated synchronized stimulations hold the potential to maintain pressures of less than the endorsed threshold via intensity fine-tuning and P_L_ control in a safe range.

A signal of protection against diaphragm atrophy also was detected. Contrary to expectations,^24^ atrophy (Tdi_exp_) was ameliorated in the intervention group. Although baseline Tdi_exp_ values were lower in the intervention group, both groups showed normal baseline Tdi_exp_ values (between 1.5 and 2.5 mm).^13^^,^^25^^,^^26^ The apparent disparity in baseline values could be attributed to the study’s limited sample size. The intervention group, despite lower baseline Tdi_exp_, demonstrated improvement. However, Tdi_exp_ does not necessarily reflect diaphragm strength, and higher Tdi_exp_ values do not guarantee improved MV weaning outcomes. The difference in Tdi_exp_ could stem from various causes. Goligher et al^27^ noted conflicting results with increased Tdi_exp_; nevertheless, pathophysiologic mechanisms require confirmation. Also, ultrasound-measured diaphragm thickness might be affected by lung volume; higher lung volume shortens the diaphragm, potentially yielding higher measured Tdi_exp_. The initial apparent rise in Tdi_exp_ might be attributed to NEPNS increasing lung volumes, a noteworthy aspect yet to be studied, possibly contributing to the overall thickness pattern divergence between groups.

In addition to MV mechanics and lung protection during bilateral transcutaneous NEPNS, it is essential to consider the intervention safety profile. Minor adverse events during stimulation had no consequences and were resolved promptly by interrupting stimulation. A single severe event, increased intracranial pressure, underwent a thorough evaluation by a multidisciplinary team of internal and independent physicians, determining it was unlikely to be linked to the procedure. Costimulation of the brachial plexus and temporary skin reddening were not classified as adverse events, being encountered in previous experiments and having posed no safety risks.^10^ O’Rourke et al^13^ reported no serious adverse events with needle phrenic nerve stimulation, although potential risks existed (infection, nerve, and vascular injury). Electrical phrenic nerve stimulation previously was evaluated in awake participants who reported discomfort during stimulation, resolved by replacing the electrodes.^28^ For transvenous temporary diaphragm neurostimulation, technical challenges in catheter insertion and positioning (unfeasibility, lack of diaphragm contraction, procedural pain) and adverse events (thrombosis at the insertion site with consequent removal of the stimulation catheter) were documented.^15^ Transvenous temporary diaphragm neurostimulation risks resemble the mechanical, thrombotic, and infectious risks of standard central venous lines.^29^ The noninvasive nature of bilateral transcutaneous NEPNS eliminates the possibility of such adverse events.

The present study had limitations. As a single-center feasibility trial, conducting larger studies with clinical end points is essential. Furthermore, because of the withdrawal of the CE certificate of the coils after the revised European Union medical device regulation, we could not assess the feasibility of more than two training sessions or the optimal training frequency. Nonetheless, we completed all the experiments for the patients receiving two stimulation sessions per day, showing promising atrophy counteraction. Because of the premature termination of the study and consequently low numbers of enrolled patients, the study includes an overrepresentation of male patients (82%); however, subsequent analysis revealed no statistically significant difference in sex distribution between the two groups. Finally, we applied stimulation manually, which may have resulted in less-than-optimal synchronization with spontaneous breathing if it was present. To address this, future perspectives include implementing an automatically synchronized stimulation system with the mechanical ventilator. PSV support and transdiaphragmatic pressures were not documented because they were not part of the prospectively recorded parameters; however, PSV levels of > 4 mbar were never used. Although a post hoc analysis was attempted for transdiaphragmatic pressures, the high prevalence of missing data and artifacts compromised the data integrity, precluding the publication of these results.

## Interpretation

Being the first, to our knowledge, implementation of bilateral transcutaneous NEPNS in the critical care setting, this study demonstrated feasibility, with a good speed of deployment (23 s) and a high realization rate exceeding 90% of planned sessions. However, TV efficacy in the 3- to 6-mL/kg IBW range was sufficiently reached only in PSV-assisted NEPNS, with a P_L_ mean of 19.0 mbar. The technique was safe, as breaths were delivered within the range of safe P_L_ swings with stimulation only, signifying a low risk of barotrauma and ventilator-induced lung injury. Elevated P_L_ mean values of 23.0 mbar were documented only during NEPNS with spontaneous breathing. Airway driving pressure remained < 15 mbar during stimulation, demonstrating a low tendency for lung stress.

## Funding/Support

This work was supported by a project grant and equipment from STIMIT AG, Biel, Switzerland.

## Financial/Nonfinancial Disclosures

The authors have reported to *CHEST* the following: S. J. S. reports grants and nonfinancial support from STIMIT AG for this work, grants and nonfinancial support from Reactive Robotics GmbH, ASP GmbH, and ESICM; grants, personal fees, and nonfinancial support from Fresenius Kabi Deutschland GmbH; grants from the Innovationsfond of The Federal Joint Committee (G-BA); personal fees from Springer Verlag GmbH for educational purposes and Advanz Pharma GmbH; and nonfinancial support from national and international societies (and their congress organizers) in the field of anesthesiology and intensive care medicine, outside the submitted work; holds stocks in small amounts from Alphabeth, Inc., Bayer AG, and Siemens AG; these holdings have not affected any decisions regarding his research or this study. A. P. holds stocks in small amounts from BioNTech SE, Taiwan Semiconductor, Sony, Pfizer, Arcutis Biotherapeutics, Inc., Sangamo Therapeutics, NIO, and Ke Holdings; these holdings have not affected any decisions regarding his research or this study. L. B. has received research grants from STIMIT, Medtronic, and Draeger and equipment from Sentec, Philips, and Fisher Paykel. None declared (A. M. G., S. K., M. A. V., B. U., J. J. G., H. G. B., N. M. C., T. N., S. W.-C.).

## Supplementary Data

Video 2

e-Online Data
