# Supplementary material for: Noninvasive Electromagnetic Phrenic Nerve Stimulation in Critically Ill Patients: A Feasibility Study
Source: Chest. 2024 Feb 24;166(3):502–10. doi: 10.1016/j.chest.2024.02.035 (PMC11443241; doi:10.1016/j.chest.2024.02.035)
Supplement: e-Online Data [file mmc3.pdf]

Online Data e-Appendix

Non-invasive electromagnetic phrenic nerve stimulation in critically ill patients – a feasibility study.

Authors:

Alessandro Panelli, MD[1]; Aline M Grimm[1]; Sven Krause, MSc[2]; Michael A Verfuß[1]; Bernhard Ulm[3,4]; Julius J Grunow, MD[1]; Hermann G Bartels[1]; Niklas M Carbon, MD [1,5]; Thomas Niederhauser, PhD[2]; Steffen Weber-Carstens MD [1]; Laurent Brochard, MD [6,7]; Stefan J Schaller, MD [1,3]

1 Charité – Universitätsmedizin Berlin, corporate member of Freie Universität Berlin and Humboldt-Universität zu Berlin, Department of Anesthesiology and Operative Intensive Care Medicine (CVK, CCM), Berlin, Germany
2 Institute For Human Centered Engineering, Bern University of Applied Sciences, Biel/Bienne, Switzerland
3 Technical University of Munich, School of Medicine, Klinikum rechts der Isar, Department of Anesthesiology and Intensive Care, Munich, Germany

4 Department of Anesthesiology, Friedrich-Alexander-Universität Erlangen-Nürnberg, Uniklinikum Erlangen, Erlangen, Germany
5 Keenan Research Centre for Biomedical Science, Li Ka Shing Knowledge Institute, Unity Health Toronto, Toronto, ON, Canada.
6 Interdepartmental Division of Critical Care, University of Toronto, Toronto, Canada.

Corresponding Author:

Univ.-Prof. Dr. med. Stefan J Schaller
Charité – Universitätsmedizin Berlin
Department of Anesthesiology and Operative Intensive Care Medicine (CVK, CCM)
Charitéplatz 1, 10117 Berlin, Germany
stefan.schaller@charite.de
ORCID: 0000-0002-6683-9584
Twitter: DrStefan2

Table of content

Table of content 2

e-Methods 3

Exclusion Criteria 3

Capture Point and Stimulation Maintenance 3

P_L, P_{occ}, P_{aw}, and Hardware Setting 4

NEPNS Stimulator Specifications 4

Methods – Blinded Diaphragm Ultrasound Assessment 4

e-Figure 1 6

e-Figure 2 7

e-Table 1 8

e-Table 2 9

e-Table 3 10

e-Table 4 11

e-Table 5 12

e-Table 6 13

Bibliography 14

e-Methods

Exclusion Criteria

Exclusion criteria were known neurological conditions affecting the motor neuron or the muscle (e.g., ALS); known paralysis of the phrenic nerve; proven or suspected spinal cord injury that contraindicates weight bearing on the spinal cord; conditions that limit diaphragm movement (e.g., high intra-abdominal pressure, ascites, obesity BMI > 35); patients with implanted cardiac support systems (pacemaker, implanted defibrillator); patients with implanted medical pumps; patients treated with medication that significantly reduces the seizure threshold; pregnant patients; patients with skin lesions, infections or strictures in throat/neck area; patients with metallic implants; patient not able to read and understand the German language.

Capture Point and Stimulation Maintenance

To identify coil positioning for stimulation (capture point, CP), the phrenic nerve was localized according to anatomical landmarks (posterior to the sternocleidomastoid muscle) by one of the ICU intensivists trained on awake volunteers. An adequate stimulation point was identified by varying the coil position on the neck surface and changing the coil angle, maintaining the same position. We assumed effective diaphragmatic contraction by administering 3 ml/kg tidal volumes based on an ideal body weight (IBW) surrogate. This aligns with recent developments in lung-protective ventilation for acute respiratory distress syndrome (ARDS), named ultra-lung-protective MV, with a predicted body weight target of 3 ml/kg.[1]

The criterion for establishing CP was met when a tidal volume of at least 3 ml/kg of ideal body weight was reached without adding PSV. Given that the maximal stimulation intensity of 50% (maximal intensity of 50% as an unsurpassable limit was stipulated by the manufacturer) did not result in tidal volumes of 3 ml/kg of ideal body weight, PSV could be added until the threshold of 3 ml/kg of ideal body weight was reached. Contractions were additionally identified directly with diaphragm ultrasound and indirectly with flow changes of the ventilator. The time to find the CP and the respective tidal volumes were documented.

The stimulation was performed using continuous positive airway pressure mode with the same positive end-expiratory pressure (PEEP) and FiO_2 previously set by the treatment team. The exclusive PMR35 dual coils (STIMIT AG, Biel, Switzerland) were used with a PowerMAG 100 clinical stimulator (Mag&More GmbH, Munich, Germany) to generate a magnetic field set just high enough to achieve a tidal volume of 3-6 ml/kg IBW; the stimulator was limited to 50% intensity.

To maintain adequate oxygenation (SpO_2) and decarboxylation ($etCO_2$) in the patients during the intervention, assisted spontaneous breathing (pressure support ventilation, PSV) mode could be used such that the ventilator supported each stimulated breath.

Stimulations were performed as a two-second-long (standard duration or reduced one-second-long) linear train with a frequency of 25 Hz. If the patient was deeply sedated and unable to generate spontaneous breaths, the stimulation rate was manually set at a physiological respiratory rate of 12 to 18 breaths per minute; however, if the patient was capable of spontaneous breathing, the stimulation was manually synchronized with their natural breathing pattern.

P_L, P_{occ}, P_{aw}, and Hardware Setting

The intervention patients were equipped with the double balloon nasogastric catheter NutriVent™ (SIDAM S.R.L., Modena, IT) combined with a recording device and V600 mechanical ventilator (both Drägerwerk AG Co. KGaA, Lübeck, DE). This hardware setting allowed the recording of airway pressure, airway flow, tidal volume, gastral pressure, and transpulmonary pressure (P_L). Airway pressure ($P_{aw\ max}$ and $P_{aw\ min}$, maximal and minimal tracheal pressure, respectively), flow, and volume were recorded and analyzed. Occlusion pressure (P_{occ}) measurements were performed three times before each stimulation session, as previously performed by Bertoni et al.[2]

NEPNS Stimulator Specifications

The exclusive PMR35 dual coils (STIMIT AG, Biel, CH) were used with a PowerMAG 100 clinical stimulator (Mag&More GmbH, Munich, DE) to perform NEPNS.

PowerMAG 100 clinical stimulator specifications: maximal output of 2400 Volt, 160 Joule, pulse length 160 µs at 100% intensity was limited to 50% intensity, i.e., each single coil had a maximal output of 0.55 Tesla and 1200 Volt.

The coils were positioned bilaterally on the patient's neck, and phrenic nerve stimulations were attempted to identify optimal coil positioning for stimulation (capture point, CP). Coil positioning is shown in our previous study, published in 2023 by Panelli and colleagues.[3]

The first operator was in charge of the stimulator and delivered stimuli to the patient's neck, positioned on the head side of the bed. The second operator performed a diaphragm ultrasound from the side of the bed, explicitly selecting the opposite side of the mechanical ventilator (see e-Figure 1).

Methods – Blinded Diaphragm Ultrasound Assessment

For the diaphragm ultrasound, the right hemidiaphragm thickness and thickening fraction were measured by placing a linear probe in the ninth or tenth intercostal space of the semi-recumbent patient between the anterior and midaxillary lines in the zone of apposition. We used a 5–13 MHz linear array transducer to display the diaphragm in brightness (B-mode) and motion mode (M-mode). After locating the diaphragm, inspiratory and end-expiratory measurements were performed in M-mode. The location of the measure was marked and further protected with transparent plastic dressing against removal.

The second operator (e-Figure 1) stored pseudo-anonymized ultrasound images and videos on a secure server within the hospital after each intervention. The second operator could not be blinded because the ultrasound recordings needed to occur during stimulation. This is due to the ultrasound image being taken simultaneously with the stimulation. The further analyses were blinded in the following fashion:

- Phase 1 (Blinding on): an independent third party (referred to as MD1) from a separate department blinded the names and dates of the files. Random identifiers were generated and assigned to the medical images by MD1 using the 'sample' function in R. This ensured that the image annotators remained blinded to patient details, with unblinding only occurring after all annotations were finalized.

- Phase 2 (Measurements): four independent image annotators (US1, US2, US3, and US4) attended a course and obtained certification from an external company specializing in diaphragm ultrasound execution and consequent measurements. The anonymized ultrasound files were assigned to two blinded image annotators (US1, US2, and US3). The annotators performed three measurements for each parameter and calculated mean values. Upon completion of the measurement, the results were saved anonymously in the shared repository. US4 was blinded and performed a fourth series of three measurements for each parameter, also anonymously generating a mean value only in case of interobserver variability above 10%.
- Phase 3 (Blinding off): MD1 removed the blinding from the dataset.
- Phase 4 (Statistical Analysis): the research group statistically analyzed unblinded data.

e-Figure 1

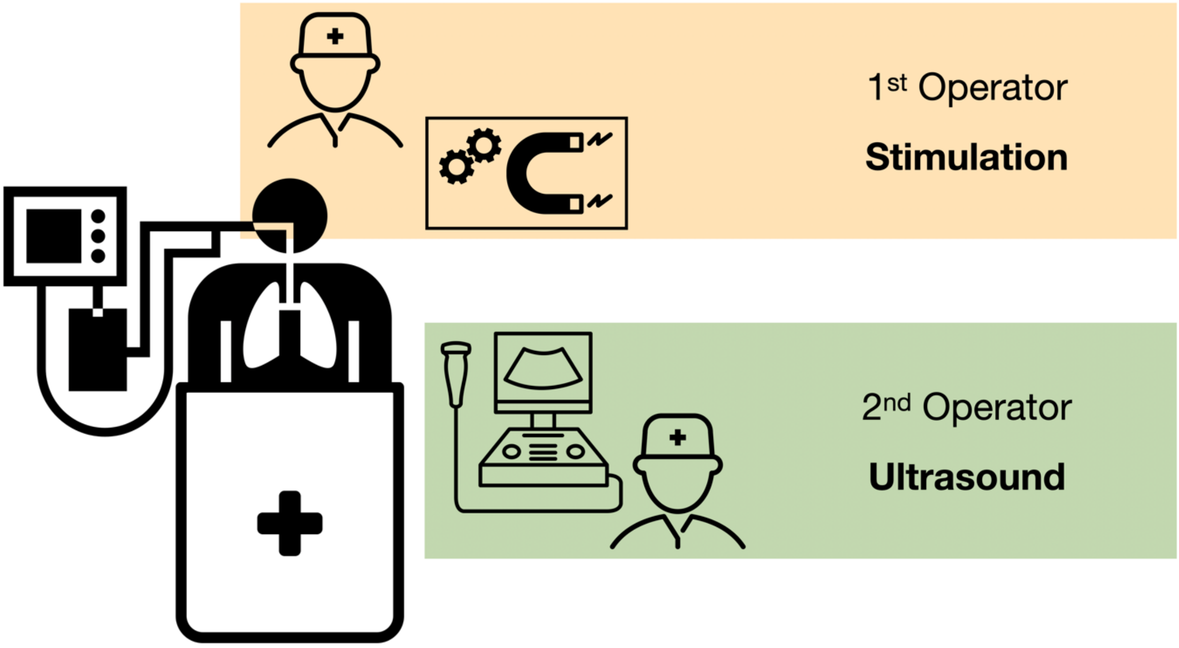

e-Figure 1. Diagram of operators and device positioning around the critically ill patient. The first operator administered NEPNS from the head side of the bed using the stimulator. Simultaneously, the second operator conducted diaphragm ultrasound on the side opposite to the mechanical ventilator.

e-Figure 2

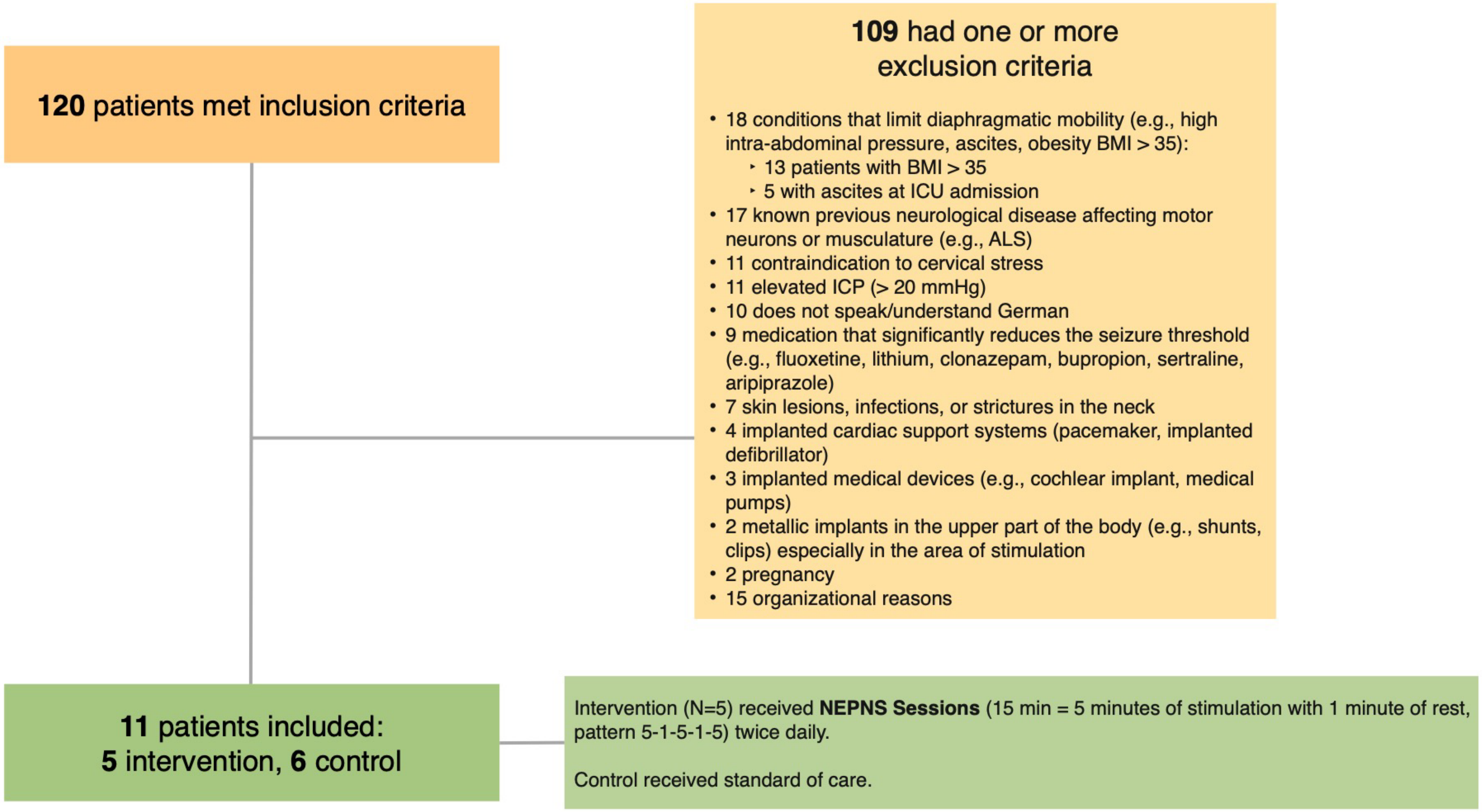

e-Figure 2. Strobe inclusion flow chart. Organizational considerations were determined by our capacity to concurrently accommodate two intervention patients or one intervention patient alongside two control patients. Due to these limitations, 15 patients were excluded despite the absence of any exclusion criteria.

e-Table 1

e-Table 1. Achieved tidal volume based on 4-8 ml/kg IBW according to current ESICM guidelines on acute respiratory distress syndrome.[4]

Stimulation Type	Tidal Volume (ml/kg IBW)	bilateral NEPNS Breaths (N)	bilateral NEPNS Breaths (%)
bilateral NEPNS only (without PSV)	< 4	1353	21.3%
	**4-8**	348	5.5%
	> 8	0	0.0%
bilateral NEPNS + PSV	< 4	1962	30.9%
	**4-8**	1750	27.5%
	> 8	324	5.1%
Spontaneous breathing + bilateral NEPNS	< 4	4	0.1%
	**4-8**	65	1.0%
	> 8	550	8.7%
**Total Stimulations (N)**		6356	100%

e-Table 2

e-Table 2. Secondary outcomes comparing intervention and control group during MV; parameters exported from the ventilator before the stimulation session.

	Intervention	Control	p-value
Lung compliance (ml / cmH2O)			
Day 1	77 [41 – 106]	49 [36 – 50]	0.37
Day 4	98 [45 – 109]	34 [19 – 49]	0.18
Day 10	47 [38 – 63]	60 [49 – 63]	0.63
Lung resistance (cmH2O / L · sec)			
Day 1	9 [7 – 11]	10 [9 – 10]	0.57
Day 4	9 [7 – 12]	7 [4 – 10]	0.49
Day 10	8 [5 – 10]	10 [8 – 10]	0.50

* A deviation of ±1 day was allowed if no measurement was available on the specified day itself.
Data are presented as median [IQR].

e-Table 3

e-Table 3. Change of diaphragm parameters comparing intervention and control group.

	Intervention	Control	p-value
**Change ΔTdi (%)**			
**Day 1 (baseline) to Day 4***	12 [-25 – 25]	16 [-18 – 21]	1
**Day 1 (baseline) to Day 10***	8 [-6 – 19]	11 [-10 – 13]	1
**Change Tdi_{exp} (mm)**			
**Day 1 (baseline) to Day 4***	0.357 [-0.547 – 1.610]	0.047 [-1.350 – 0.336]	0.41
**Day 1 (baseline) to Day 10***	0.926 [0.398 – 1.620]	-0.731 [-1.086 – -0.398]	0.036
**Change Tdi_{insp} (mm)**			
**Day 1 (baseline) to Day 4***	0.751 [-0.239 – 1.524]	0.120 [-0.494 – 0.882]	0.786
**Day 1 (baseline) to Day 10***	1.289 [0.427 – 1.980]	-0.499 [-2.099 – -0.311]	0.036
**Change in DE (mm)**			
**Day 1 (baseline) to Day 4***	n/a[#]	n/a[$]	n/a
**Day 1 (baseline) to Day 10***	0.109**	n/a[$]	n/a

* A deviation of ±1 day was allowed if no measurement was available on the specified day itself.
** p-value was calculated using Wilcoxon-test
[#] values for DE on day 4 not available, due to difficult echography window.
[$] values for DE not prospectively planned for control group.
Data are presented as median [range].
n/a not applicable.

e-Table 4

e-Table 4. Exploratory clinical outcomes.

Variables	Intervention<br><br>n = 5	Control<br><br>n = 6	All<br><br>n = 11
Ventilation duration	13 [10.8-33.5]	29.5 [8-33]	21 [10-33]
ICU LOS	15 [13.2-33.5]	15.5 [11-29.3]	15 [11-36]
Hospital LOS	15 [13.5-35]	33.5 [22-46]	23 [14-46]
ICU mortality	2 (40%)	2 (33%)	4 (36%)
Hospital mortality	3 (60%)	2 (33%)	8 (46%)

Data are presented as n (%) and median [IQR]. There are no group differences in the variables presented.

e-Table 5

e-Table 5. Summary of adverse events during stimulation sessions possibly related to the intervention.

**Variable**	**n (%)**<br>**n = 92**
AE Desaturation < 93%	10 (11)
AE Hypertension, MAP > 90 mmHg	2 (2)
AE Hypotension, MAP < 60 mmHg	2 (2)
AE self-limiting extrasystoles	1 (1)
AE respiratory acidosis (pCO_2 > 50 mmHg and pH < 7.2)	1 (1)
SAE ICP increase	1 (1)
**At least 1 AE in a stimulation session**	**17 (18)**

AE, adverse events; ICP, intracranial pressure; MAD, mean arterial pressure; pCO2, arterial partial pressure of carbon dioxide; SAE, serious adverse events.

e-Table 6

e-Table 6. Ventilation modes, PIP, PEEP, FiO2 for the 11 included patients over time (10 days). Ventilator free days are signalized in green.

Day	1	2	3	4	5	6	7	8	9	10	Ventilator Free Days
Patient 1<br>Intervention	PC-AC<br>30% FiO2<br>14 PIP<br>8 PEEP	PC-AC<br>25% FiO2<br>17 PIP<br>8 PEEP	PC-AC<br>30% FiO2<br>16 PIP<br>8 PEEP	CPAP<br>30% FiO2<br>8 PEEP	SPN-CPAP<br>30% FiO2<br>14 PIP<br>8 PEEP	PC-BIPAP<br>30% FiO2<br>16 PIP<br>8 PEEP	PC-BIPAP<br>30% FiO2<br>16 PIP<br>8 PEEP	PC-BIPAP<br>30% FiO2<br>17 PIP<br>8 PEEP	PC-BIPAP<br>30% FiO2<br>23 PIP<br>8 PEEP	CPAP<br>30% FiO2<br>8 PEEP	2/10 (20%)
Patient 2<br>Control	PC-BIPAP<br>35% FiO2<br>22 PIP<br>12 PEEP	PC-BIPAP<br>45% FiO2<br>24 PIP<br>12 PEEP	PC-BIPAP<br>35% FiO2<br>22 PIP<br>12 PEEP	ASV<br>30% FiO2<br>12 PEEP	ASV<br>30% FiO2<br>12 PEEP	PC-BIPAP<br>35% FiO2<br>19 PIP<br>10 PEEP	PC-BIPAP<br>30% FiO2<br>19 PIP<br>10 PEEP	PC-BIPAP<br>30% FiO2<br>20 PIP<br>10 PEEP	PC-BIPAP<br>30% FiO2<br>19 PIP<br>10 PEEP	PC-BIPAP<br>30% FiO2<br>19 PIP<br>10 PEEP	2/10 (20%)
Patient 3<br>Control	PCMV<br>50% FiO2<br>40 PIP<br>14 PEEP	PCMV<br>60% FiO2<br>41 PIP<br>14 PEEP	PC-BIPAP<br>50% FiO2<br>38 PIP<br>16 PEEP	PCMV<br>60% FiO2<br>36 PIP<br>16 PEEP							0/4 (0%)
Patient 4<br>Intervention	PC-AC<br>35% FiO2<br>25 PIP<br>15 PEEP	PC-AC<br>60% FiO2<br>23 PIP<br>13 PEEP	PC-AC<br>50% FiO2<br>23 PIP<br>13 PEEP	CPAP<br>40% FiO2<br>19 PIP<br>10 PEEP	PC-AC<br>50% FiO2<br>20 PIP<br>14 PEEP	PC-AC<br>50% FiO2<br>20 PIP<br>14 PEEP	PC-AC<br>75% FiO2<br>28 PIP<br>16 PEEP	PC-AC<br>70% FiO2<br>26 PIP<br>15 PEEP	PC-AC<br>80% FiO2<br>22 PIP<br>14 PEEP	PC-BIPAP<br>70% FiO2<br>23 PIP<br>12 PEEP	1/10 (10%)
Patient 5<br>Intervention	PC-BIPAP<br>70% FiO2<br>23 PIP<br>12 PEEP	PC-BIPAP<br>70% FiO2<br>26 PIP<br>14 PEEP	PC-BIPAP<br>65% FiO2<br>26 PIP<br>16 PEEP	PC-BIPAP<br>40% FiO2<br>24 PIP<br>14 PEEP	PC-BIPAP<br>100% FiO2<br>24 PIP<br>16 PEEP	PC-BIPAP<br>60% FiO2<br>23 PIP<br>16 PEEP	PC-BIPAP<br>85% FiO2<br>26 PIP<br>16 PEEP	PC-BIPAP<br>70% FiO2<br>23 PIP<br>17 PEEP	PC-BIPAP<br>65% FiO2<br>23 PIP<br>17 PEEP	PC-BIPAP<br>40% FiO2<br>32 PIP<br>17 PEEP	0/10 (0%)
Patient 6<br>Control	PC-BIPAP<br>35% FiO2<br>23 PIP<br>14 PEEP	O2-<br>Insufflation<br>4 L/min	PC-AC<br>80% FiO2<br>11 PIP<br>8 PEEP	PC-AC<br>45% FiO2<br>42 PIP<br>8 PEEP	PC-AC<br>35% FiO2<br>38 PIP<br>14 PEEP	PC-AC<br>30% FiO2<br>37 PIP<br>12 PEEP	PC-AC<br>30% FiO2<br>44 PIP<br>12 PEEP	PC-AC<br>30% FiO2<br>35 PIP<br>12 PEEP	CPAP<br>25% FiO2<br>10 PEEP	CPAP<br>30% FiO2<br>10 PEEP	3/10 (30%)
Patient 7<br>Control	ASV<br>31% FiO2<br>8 PEEP	ASV<br>46% FiO2<br>8 PEEP	DuoPAP<br>36% FiO2<br>15 PIP<br>5 PEEP	DuoPAP<br>51% FiO2<br>15 PIP<br>5 PEEP	DuoPAP<br>36% FiO2<br>14 PIP<br>5 PEEP	DuoPAP<br>31% FiO2<br>14 PIP<br>5 PEEP	DuoPAP<br>31% FiO2<br>21 PIP<br>5 PEEP	DuoPAP<br>31% FiO2<br>16 PIP<br>8 PEEP	DuoPAP<br>31% FiO2<br>15 PIP<br>8 PEEP	DuoPAP<br>31% FiO2<br>15 PIP<br>7 PEEP	2/10 (20%)
Patient 8<br>Intervention	PC-BIPAP<br>50% FiO2<br>28 PIP<br>15 PEEP	PC-BIPAP<br>40% FiO2<br>35 PIP<br>15 PEEP	PC-BIPAP<br>85% FiO2<br>28 PIP<br>15 PEEP	PC-BIPAP<br>50% FiO2<br>28 PIP<br>15 PEEP	PC-BIPAP<br>30% FiO2<br>27 PIP<br>15 PEEP	PC-BIPAP<br>40% FiO2<br>28 PIP<br>13 PEEP	PC-BIPAP<br>45% FiO2<br>26 PIP<br>14 PEEP	PC-BIPAP<br>30% FiO2<br>26 PIP<br>14 PEEP	PC-BIPAP<br>50% FiO2<br>25 PIP<br>12 PEEP	CPAP<br>30% FiO2<br>9 PEEP	1/10 (10%)
Patient 9<br>Intervention	PC-BIPAP<br>30% FiO2<br>12 PIP<br>7 PEEP	PC-BIPAP<br>35% FiO2<br>14 PIP<br>7 PEEP	PC-BIPAP<br>35% FiO2<br>13 PIP<br>7 PEEP	PC-BIPAP<br>35% FiO2<br>13 PIP<br>7 PEEP	PC-BIPAP<br>40% FiO2<br>13 PIP<br>7 PEEP	PC-BIPAP<br>40% FiO2<br>12 PIP<br>7 PEEP	PC-BIPAP<br>40% FiO2<br>13 PIP<br>7 PEEP	PC-BIPAP<br>40% FiO2<br>15 PIP<br>7 PEEP	PC-BIPAP<br>40% FiO2<br>16 PIP<br>7 PEEP	PC-BIPAP<br>40% FiO2<br>14 PIP<br>7 PEEP	0/10 (0%)
Patient 10<br>Control	PC-BIPAP<br>40% FiO2<br>15 PIP<br>7 PEEP	PC-BIPAP<br>30% FiO2<br>17 PIP<br>7 PEEP									0/2 (0%)
Patient 11<br>Control	PC-BIPAP<br>30% FiO2<br>23 PIP<br>10 PEEP	PC-BIPAP<br>30% FiO2<br>25 PIP<br>10 PEEP	PC-BIPAP<br>30% FiO2<br>25 PIP<br>12 PEEP	PC-BIPAP<br>30% FiO2<br>24 PIP<br>12 PEEP	PC-BIPAP<br>40% FiO2<br>28 PIP<br>12 PEEP	PC-BIPAP<br>40% FiO2<br>27 PIP<br>10 PEEP	PC-BIPAP<br>40% FiO2<br>25 PIP<br>10 PEEP	PC-BIPAP<br>45% FiO2<br>22 PIP<br>10 PEEP	PC-BIPAP<br>35% FiO2<br>23 PIP<br>10 PEEP	PC-BIPAP<br>30% FiO2<br>27 PIP<br>10 PEEP	0/10 (0%)

Bibliography

1. Abrams D, Agerstrand C, Beitler JR, et al. Risks and Benefits of Ultra-Lung-Protective Invasive Mechanical Ventilation Strategies with a Focus on Extracorporeal Support. *Am J Respir Crit Care Med.* 2022;205(8):873-882.
2. Bertoni M, Telias I, Urner M, et al. A novel non-invasive method to detect excessively high respiratory effort and dynamic transpulmonary driving pressure during mechanical ventilation. *Crit Care.* 2019;23(1):346.
3. Panelli A, Bartels HG, Krause S, et al. First non-invasive magnetic phrenic nerve and diaphragm stimulation in anaesthetized patients: a proof-of-concept study. *Intensive Care Med Exp.* 2023;11(1):20.
4. Grasselli G, Calfee CS, Camporota L, et al. ESICM guidelines on acute respiratory distress syndrome: definition, phenotyping and respiratory support strategies. *Intensive Care Med.* 2023.
